# Evaluation of evidence of prevention and management of facial pressure injuries in medical staff

**DOI:** 10.1002/nop2.1543

**Published:** 2022-12-11

**Authors:** Honghong Su, Qian Lv, Yue Kong, Huiling Zeng, Wenguang Zhou, Fangfang Zhu, Baoling Xu, Qijun Zhou

**Affiliations:** ^1^ Nursing College Fujian University of Traditional Chinese Medicine Fuzhou China; ^2^ Teaching and Research Department Fuzong Clinical Medical College of Fujian Medical University (The 900th Hospital of Joint Logistic Support Force, PLA) Fuzhou China; ^3^ Department of Equipment Chenggong Hospital of Xiamen University (the 73th Group Military Hospital of People's Liberation Army) Xiamen China; ^4^ Medical College Qiqihar Medical University Qiqihar China

**Keywords:** COVID‐19, evidence‐based nursing, facial pressure injuries, medical staff, personal protective equipment

## Abstract

**Aim:**

This systematic review evaluated the quality of evidence for the prevention and management of facial pressure injuries in medical staff.

**Design:**

This review was presented in accordance with the Preferred Reporting Items for Systematic Reviews and Meta‐Analyses guidelines.

**Methods:**

We retrieved the relevant studies from 19 databases. Using the literature evaluation standards and evidence grading system of the Australian Joanna Briggs Institute Evidence‐Based Health Care Center, we evaluated the quality of the literature encompassing different types of research and assessed their levels of evidence.

**Results:**

A total of 13 studies were included, including seven expert consensuses, two recommended practices, one clinical decision, one best practice information booklet, one systematic review and one randomized controlled trial. In the end, 31 best evidence were summarized, including skin cleaning and care, PPE placement and movement, reasonable use of dressings, treatment measures and education and training.

## 1 INTRODUCTION

In December 2019, the novel coronavirus pneumonia outbreak occurred. On 12 January 2020, the World Health Organization (WHO) named the disease coronavirus disease 2019 (COVID‐19) (Jin et al., 2020), and on 30 January 2020, the WHO declared it a public health emergency of international concern (Cucinotta & Vanelli, 2020). Droplet transmission and contact transmission through the respiratory tract are currently believed to be the main transmission routes of COVID‐19 (General Office of National Health Committee, 2021). In terms of the age distribution of the infected individuals, not all age groups showed COVID‐19 resistance (Khan et al., 2020). Patients with COVID‐19 and individuals in close contact with infected yet asymptomatic individuals are high‐risk groups of COVID‐19 (Epidemiology Working Group for NCIP Epidemic Response & Chinese Center for Disease Control and Prevention, 2020; Wang et al., 2020). The medical staff attending to COVID‐19 patients are at high risk of infection as they have to closely interact with the patients (Kluytmans‐van den Bergh et al., 2020; Kua et al., 2021). Therefore, to minimize the risk of contracting the virus, doctors, nurses and other medical professionals around the world need to use personal protective equipment (PPE; Mahmood et al., 2020).

In the process of using masks, goggles and face shields to avoid contracting the disease, the use of PPE entails close contact with the skin, and to prevent cross‐infection, the removal of protective equipment needs to be minimized, which results in prolonged use of the protective equipment and sustained pressure on local skin (European Pressure Ulcer Advisory Panel (EPUAP), 2021). Under such a demanding work environment requiring prolonged use of face masks coupled with increased workload and mental stress, the skin sweats a lot, resulting in a humid environment inside the mask (Bischoff et al., 2019). The skin is the first line of defence against the environment, and it is repeatedly affected by physical factors (sustained pressure, tension and friction) and chemical factors (humidity), and because of these factors, the tolerance of the skin changes, causing skin resistance to decline (Sivamani et al., 2003). These factors are directly associated with the occurrence of pressure ulcers and friction injuries (Schwartz et al., 2018) and eventually lead to facial pressure injuries. The most commonly affected parts are the nasal bridge, cheeks, forehead and ears (Darlenski & Tsankov, 2020). The results of a multi‐centre cross‐sectional survey conducted by Jiang et al. (2020) revealed that the overall incidence of skin damage caused by PPE among medical staff was 42.8%. Facial pressure injuries are usually regarded as mild irritation and are often overlooked (LeBlanc et al., 2021). However, it is worth noting that even minor skin irritation can increase the risk of infection in medical staff because skin irritation may cause people to subconsciously touch their faces when they are not wearing PPE (Gefen & Ousey, 2020a; Kantor, 2020). Furthermore, facial pressure injuries can also cause discomfort and local skin conditions, such as erythema, indentation, a stinging sensation, reduced facial comfort, localized warmth and weakened sense of touch, and occasional skin breakage (Wang & Parish, 2019).

Fortunately, if appropriate measures are taken, the facial and ear injuries that medical staff are most vulnerable to can also be prevented. A parallel double‐arm randomized clinical trial compared the use of foam and extra‐thin hydrocolloid dressing by medical staff working at the forefront of the fight against COVID‐19 to prevent PPE‐induced facial stress injuries (Gasparino et al., 2021). The results showed that foam and extra‐thin hydrocolloid dressing could effectively prevent PPE‐induced facial stress injuries (Gasparino et al., 2021). The National Pressure Injury Advisory Panel recommends that medical staff clean the skin before and after wearing PPE, treat PPE‐related pressure injury and reduce the pressure caused by PPE (European Pressure Ulcer Advisory Panel (EPUAP), 2021). A self‐controlled study showed that the use of a hydrocolloid dressing combined with 3 M Cavilon No‐Sting Barrier Film for facial skincare can effectively reduce the incidence of facial pressure injuries in medical staff (Zhang et al., 2021). At present, several studies have reported on the prevention and management of facial pressure injuries in medical staff. However, uniform standards for the prevention and management of facial pressure injuries in medical staff remain to be established.

This study was aimed at (1) providing references for clinical practice in the prevention and management of facial pressure injuries in medical staff, (2) reducing the incidence of such injuries and (3) improving the facial comfort of medical staff. To this end, we collected and evaluated the evidence obtained through evidence‐based nursing methods and summarized the findings.

## 2 METHODS

### 2.1 Establishment of the problem

We used the problem development tool of the population, intervention, professional, outcome, setting and type of evidence (PIPOST) model of the Joanna Briggs Institute (JBI) Evidence‐Based Health Care Center to construct evidence‐based problems. The first ‘P’ (population) is the target population for the application of evidence. This includes medical personnel working at the frontlines in the fight against COVID‐19, those working in fever clinics and those who go out for batch nucleic acid testing or to infection wards and need to wear protective equipment. ‘I’ (intervention) is the recommended intervention. This includes the prevention and management of facial pressure injuries caused by the use of protective equipment. The second ‘P’ (professional) is the implementer of evidence application, that is, medical staff. ‘O’ (outcome) is the outcome indicator, which is the alleviation of facial pressure injury and restoration of facial comfort. ‘S’ (setting) is the evidence application site, that is, anti‐epidemic frontline, fever clinic, nucleic acid testing site or infection ward. ‘T’ (type of evidence) is the type of evidence resource, which includes guidelines, expert consensus, clinical decisions, recommended practices, best practice information booklets, systematic reviews, evidence summaries and randomized controlled trials (RCTs).

### 2.2 Evidence sources and retrieval strategies

According to the ‘6 S’ pyramid evidence model, we searched the following databases: British Medical Journal Best Practice, UpToDate, the Guidelines International Network, the National Institute for Health and Care Excellence, the Scottish Intercollegiate Guidelines Network, the Registered Nurses Association of Ontario, the European Pressure Ulcer Advisory Panel, the New Zealand Wound Care Society, the American Academy of Dermatology Association, the European Academy of Dermatology Association, the Cochrane Library, JBI, the Cumulative Index to Nursing and Allied Health Literature, EMBASE, SinoMed, PubMed, the China National Knowledge Infrastructure database, Wanfang databases and the Chinese Scientific Journal Database. The search keywords used were as follows: ‘pressure ulcer’ ‘device‐related pressure ulcer’, ‘MDRPI’, ‘MDRPU’, ‘facial skin injury’, ‘PPE‐related tissue damage’, ‘COVID‐19‐related pressure injury’, ‘skin lesion’, ‘facial injury’, ‘personal protective equipment’, ‘COVID‐19’, ‘ “SARS‐CoV‐2,” ‘Coronavirus Disease 2019’ and ‘COVID‐19 Pandemic’. The article types searched were guidelines, expert consensus, clinical decisions, recommended practices, best practice information booklets, systematic reviews, evidence summaries and RCTs. The retrieval period was from 1 January 2010 to 23 October 2021.

### 2.3 Study inclusion and exclusion criteria

The inclusion criteria were as follows: (1) the research object was medical staff; (2) the research was aimed at the prevention and management of facial pressure injuries; (3) the types of reports included were guidelines, expert consensus, clinical decisions, practice recommendations, best practice information booklets, systematic reviews, evidence summaries and RCT findings; and (4) the language was Chinese or English.

The exclusion criteria were as follows: (1) duplicate publications; (2) studies with original text unavailable; and (3) studies with unqualified quality evaluation.

### 2.4 Literature quality evaluation

#### 2.4.1 Literature quality evaluation process

The literature quality evaluation was independently completed by two authors. For training, these authors had enrolled into and completed the Evidence‐Based Nursing Practitioner Training Class of the JBI Evidence‐based Nursing Cooperation Center of Fudan University. If there was a disagreement, the two authors tried to resolve their disagreements by discussion, and if they could still not reach a consensus, they invited evidence‐based nursing experts and evidence‐based training instructors to make a judgement after thorough discussion.

#### 2.4.2 Literature quality evaluation method

##### Evaluation of guidelines

The quality evaluation standard of guidelines adopted the Appraisal of Guidelines for Research and Evaluation Instrument (AGREE II; Brouwers et al., 2010) for quantitative scoring. The scale had a total of six areas, 23 main items, and two additional overall evaluation items. Each item was evaluated on a scale of 1 to 7 (1 = strong disagreement, 7 = strong agreement). According to the scores in each field, the recommended level was evaluated.

##### Evaluation of expert consensus

The quality evaluation standard of the expert consensus adopted the corresponding evaluation standard of the Australian JBI Evidence‐based Health Care Center (2016; Tufanaru et al., 2020). There were six items in total. The evaluation results were divided into ‘Yes’, ‘No’, ‘Unclear’ and ‘Not applicable’.

##### Evaluation of systematic reviews

The quality evaluation standard of systematic reviews adopted the corresponding evaluation standard of the Australian JBI Evidence‐based Health Care Center (2016; Aromataris et al., 2015). There were 11 items in total. The evaluation options were divided into ‘Yes’, ‘No’, ‘Unclear’ and ‘Not applicable’.

##### Evaluation of randomized controlled trials

The quality evaluation standard of randomized controlled trial reports adopted the corresponding evaluation standard of the Australian JBI Evidence‐Based Health Care Center (2016; Tufanaru et al., 2020). There were 13 items in total, and the evaluation options were divided into ‘Yes’, ‘No’, ‘Unclear’ and ‘Not applicable’.

##### Evaluation of clinical decisions, practice recommendations, best practice information booklets, recommended practices and evidence summaries

For the quality evaluation of these literatures, we traced the original literature on which these evidence were based and selected the corresponding evaluation standards of the Australian JBI Evidence‐based Health Care Center (Tufanaru et al., 2020) according to the type of literature.

### 2.5 Evidence summaries, classifications and recommendation levels

We screened the included studies individually, extracted the evidence based on the research question and then reviewed the evidence. We used the ‘2014 JBI Evidence Pre‐grading and Evidence Recommendation Grade System’ (Aromataris et al., 2015) to evaluate and grade the included evidence. According to the validity, feasibility, suitability and clinical significance of the evidence, the JBI evidence recommendation strength grading principle was combined to clarify the recommendation level of the evidence. The system divided the evidence level into five levels, and the recommendation opinions are divided into two recommendations (A and B).

## 3 RESULTS

### 3.1 Included studies

**FIGURE 1:**
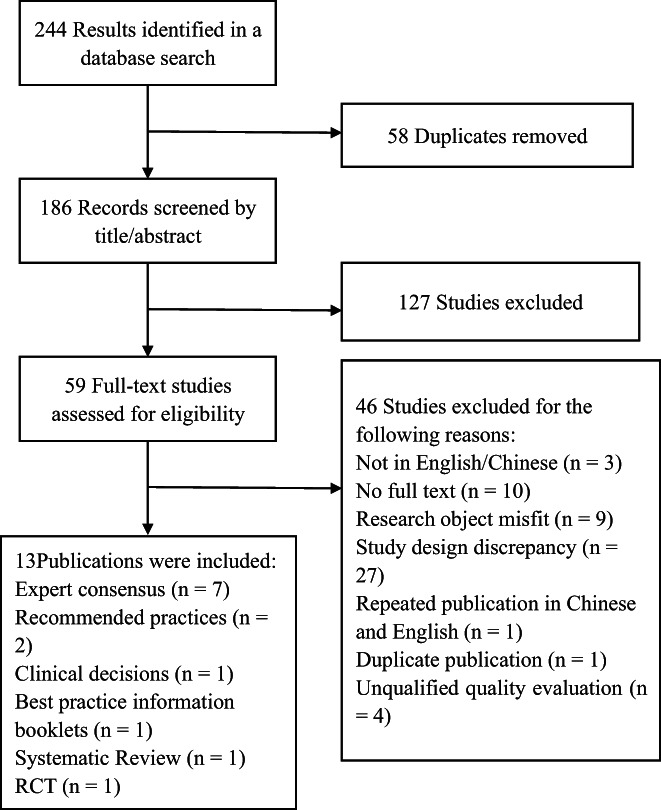
Flow diagram of study search and screen.

The retrieval flow chart is shown in Figure 1. A total of 244 studies were retrieved and imported into the ENDNOTE v9.0 software. After review, 58 duplicate studies were removed, and 127 studies were removed after reading the titles and abstracts and full texts were checked for the remaining 59 studies. After reading the full texts of these studies, we excluded studies that did not qualify on quality evaluation, studies that were inconsistent in research types and research objects and repeated publications. Finally, 13 studies were included, including seven expert consensuses, two recommended practices, one clinical decision, one best practice information booklet, one systematic review and one RCT. The general information of the included studies is shown in Table 1.

**TABLE 1:** General information included in the studies

Inclusion study	Years	Evidence source	Evidence theme	Evidence type
EPUAP	2020	EPUAP	NPIAP position statements on preventing injury with N95 masks	Expert consensus
ALeBlanc	2020	NSWOCC	Prevention and management of personal protective equipment skin injury: Update 2020	Expert consensus
Alves	2020	EPUAP	PREPI | COVID19. Prevention of skin lesions caused by personal protective equipment (face masks, respirators, visors and protection glasses)	Expert consensus
Bruce	2020	AAD	Preventing and treating occupationally induced dermatologic conditions during COVID‐19	Expert consensus
EADV	2020	EADV	Prevention of pressure injuries due to face masks during COVID‐19 pandemic	Expert consensus
Steven	2021	UpToDate	COVID‐19: Cutaneous manifestations and issues related to dermatologic care	Clinical decisions
Coyer	2020	CINAHL	Maintaining skin health and integrity for staff wearing personal protective equipment for prolonged periods: a practical tip sheet	Best practice information booklets
Gasparino	2020	Cochrane	Prophylactic dressings in the prevention of pressure ulcer related to the use of personal protective equipment by health professionals facing the COVID‐19 pandemic: A randomized clinical trial	RCT
Gefen	2020	Embase	Update to device‐related pressure ulcers: SECURE prevention. COVID‐19, face masks and skin damage	Expert consensus
Padula	2021	PubMed	Best‐practices for preventing skin injury beneath personal protective equipment during the COVID‐19 pandemic: A position paper from the National Pressure Injury Advisory Panel	Recommended practices
Yu	2021	Embase	COVID‐19 related pressure injuries in patients and personnel: A systematic review	Systematic review
Chen	2020	VIP	Urgent recommendation protective measures of West China Hospital for medical personnel to prevent device‐related pressure injuries in 2019‐nCoV epidemic situation	Recommended practices
Yan	2020	SinoMed	Consensus of Chinese experts on protection of skin and mucous membrane barrier for health professions fighting against coronavirus disease 2019	Expert consensus

Abbreviations: AAD, American Academy of Dermatology Association; EADV, European Academy of Dermatology Association; EPUAP, European Pressure Ulcer Advisory Panel; NSWOCC, New Zealand Wound Case Society.

### 3.2 Evaluation of publication quality

#### 3.2.1 Quality evaluation of expert consensus

Items were from the evaluation standard of the Australian JBI Evidence‐Based Health Care Center (2016). We included seven expert consensuses (Alves et al., 2020; American Academy of Dermatology Association (AAD), 2021; European Academy of Dermatology Association (EADV), 2021; European Pressure Ulcer Advisory Panel (EPUAP), 2021; Gefen & Ousey, 2020b; LeBlanc et al., 2021; Yan et al., 2020). Among them, in six expert consensuses, (Alves et al., 2020; European Academy of Dermatology Association (EADV), 2021; European Pressure Ulcer Advisory Panel (EPUAP), 2021; Gefen & Ousey, 2020b; LeBlanc et al., 2021; Yan et al., 2020) item 6 in their evaluation (*Is any incongruence with the literature/sources logically defended*?) was evaluated as ‘No’, and the remaining items were evaluated as ‘Yes’. In the remaining one expert consensus, (American Academy of Dermatology Association (AAD), 2021) items 5 (*Is there a reference to the extant literature*?) and 6 (*Is any incongruence with the literature/sources logically defended*) were evaluated as ‘No’, and the remaining items were evaluated as ‘Yes’.

#### 3.2.2 Quality evaluation of the systematic review

Items were from the evaluation standard of the Australian JBI Evidence‐based Health Care Center (2016). We included one systematic review (Yu et al., 2021), for which item 8 (*Were the methods used to combine studies appropriate*?) was evaluated as ‘Not applicable’, and item 9 (*Was the likelihood of publication bias assessed*?) was evaluated as ‘No’. The remaining items evaluated as ‘Yes’.

#### 3.2.3 Quality evaluation of the RCT

Items were from the evaluation standard of the Australian JBI Evidence‐based Health Care Center (2016). A randomized controlled study (Gasparino et al., 2021) was included, and for items 7 (*Were treatment groups treated identically other than the intervention of interest*?), 8 (*Was follow up complete and if not, were differences between groups in terms of their follow up adequately described and analysed*?), and 11 (*Were outcomes measured in a reliable way*?), the evaluation was ‘Unclear’. For item 9 (*Were participants analysed in the groups to which they were randomized*?), the evaluation was ‘No’, and for the remaining items, the evaluation was ‘Yes’.

#### 3.2.4 Quality evaluation of recommended practices, clinical decisions and best practice information booklets

Items were from the evaluation standard of the Australian JBI Evidence‐based Health Care Center (2016). In this study, we included two recommended practices (Chen et al., 2020; Padula et al., 2021), one clinical decision (Steven & Esther, 2021), and one best practice information booklet (Coyer et al., 2020), and upon tracing the original literature of evidence, we obtained one systematic review (Cai et al., 2019), one expert consensus (Edsberg et al., 2016), one RCT (Towfigh et al., 2008), one quasi‐experimental study (Tomas et al., 2015) and one cohort study (Visscher et al., 2015). Regarding the quality evaluation results of the systematic review, item 9 (*Was the likelihood of publication bias assessed*?) was evaluated as ‘No’, and the rest of the items were evaluated as ‘Yes’. The quality evaluation result for the expert consensus item 6 (*Is any incongruence with the literature/sources logically defended*?) was ‘No’, and the rest of the items were evaluated as ‘Yes’. The quality evaluation result of the RCT for item 5 (*Were those delivering treatment blind to treatment assignment*?) was ‘No’, and the rest of the items were evaluated as ‘Yes’. The quality evaluation result of the quasi‐experimental study was ‘Yes’ for all items. The quality evaluation results of the cohort study for items 9 (*Was follow up complete, and if not, were the reasons to loss to follow up described and explored*?) and 10 (*Were strategies to address incomplete follow up utilized*?) were ‘Unclear’. The remaining items were evaluated as ‘Yes’.

### 3.3 Synthesis of evidence

We summarized 31 pieces of evidence in five areas: skin cleaning and care, PPE placement and movement, reasonable use of dressings, treatment measures, and education and training. There were two pieces of Grade I evidence, 1 piece of Grade II evidence, 1 piece of Grade III evidence and 27 pieces of Grade V evidence. There were 16 strongly recommended items and 15 weakly recommended items. See Table 2 for details.

**TABLE 2:** Summary of the best evidence for the prevention and management of facial pressure injuries in medical staff

Type of evidence	Serial number	Evidence	Level of evidence	Recommendation level
Skin cleaning and care	1	Maintain good skin care practices. Keep the skin clean and appropriately hydrated. European Pressure Ulcer Advisory Panel (EPUAP) (2021), Gefen and Ousey (2020b)	V	A
	2	Before and after wearing PPE, practice proper hand washing. To remove oil, dirt, bacteria and viral surface contamination from the face and neck, thoroughly cleanse these areas using a pH‐balanced cleanser, physiological saline solution, or soap and water solution. McArthur et al. (2015), Alves et al. (2020), American Academy of Dermatology Association (AAD) (2021), Coyer et al. (2020), Gefen and Ousey (2020b), Padula et al. (2021)	V	A
	3	Do not rub the facial skin as this may increase tissue damage. McArthur et al. (2015)	V	A
	4	Monitor whether the facial skin is damaged, and pay attention to the damaged parts; if any damage is identified, record the damaged parts and the scope of damage. McArthur et al. (2015), Chen et al. (2020), Padula et al. (2021),	V	A
	5	Dry the face well and then apply a moisturizing cream. McArthur et al. (2015), Alves et al. (2020), Chen et al. (2020), Coyer et al. (2020), Gefen and Ousey (2020b)	I	A
	6	Consider using hyperoxygenated fatty acids (AGH) or a cream based on acrylate polymer and/or dimethicone (longer durability). McArthur et al. (2015), Alves et al. (2020), Padula et al. (2021)	V	B
	7	Moisturizing cream and/or barrier protectors should be applied to regions of greater surface contact (nose bridge, cheek bones, chin, behind the ears and points on the forehead and scalp) with PPE. McArthur et al. (2015), Alves et al. (2020), Padula et al. (2021)	V	A
	8	Moisturizing cream should be gently applied to the respective areas (ears, forehead, nose and malar areas). Alves et al. (2020)	V	A
	9	Application of a liquid skin sealant/protectant on skin surfaces that will be in contact with the mask may help prevent friction injuries without interfering with the fit of the N95 mask. American Academy of Dermatology Association (AAD; 2021), European Academy of Dermatology Association (EADV; 2021), Gefen and Ousey (2020b), European Pressure Ulcer Advisory Panel (EPUAP; 2021), Padula et al. (2021), Yan et al. (2020)	I	A
	10	Cautiously apply liquid skin sealants/protectants to avoid contact with eyes and mucous membranes. European Pressure Ulcer Advisory Panel (EPUAP; 2021), Padula et al. (2021)	V	B
	11	Before PPE application, ensure that the applied moisturizer has been allowed to dry to form a film such that it does not affect the seal of or compromise the efficacy of PPE. McArthur et al. (2015), Alves et al. (2020), American Academy of Dermatology Association (AAD; 2021), European Pressure Ulcer Advisory Panel (EPUAP; 2021), Gefen and Ousey (2020b), Padula et al. (2021)	V	B
	12	The use of petrolatum or mineral oil is not advised as a skin prep; these products can cause the mask to slip out of place and require frequent reapplication, which would involve frequently touching the face. European Pressure Ulcer Advisory Panel (EPUAP; 2021); Padula et al. (2021)	V	B
	13	Healthcare professionals need to optimize hydration and nutrition to ensure good skin health and a balanced physiological response. McArthur et al. (2015)	V	B
	14	Once safely inside their homes, those who have had to wear a PPE for long periods of time can apply a cream or an ointment, such as petrolatum jelly, to areas where the skin has been compromised, particularly the forehead, cheeks and bridge of the nose American Academy of Dermatology Association (AAD; 2021))	V	B
PPE placement and movement	15	Wear appropriate PPE, and adjust the device to the shape of your nose/face before wearing PPE. Ensure that you do not feel discomfort at any specific point of contact between the skin and the device and that there is no unnecessary pressure/tension at the points of contact (ears, forehead, nose and cheek). McArthur et al. (2015), Alves et al. (2020), Chen et al. (2020), Yan et al. (2020)	V	A
	16	Remove the mask from your face for 15 min at 2‐h intervals outside of areas of patient contact. If this time frame is not practically possible, try to lift the mask by the sides for 5 min every 2 h with clean hands. Any pressure relief provided to the skin and soft tissue will be beneficial. European Pressure Ulcer Advisory Panel (EPUAP; 2021), Gefen and Ousey (2020b), Padula et al. (2021)	III	A
	17	If masks or other parts of PPE feel uncomfortable, they should be removed as soon as possible in a safe area, and the skin should be checked for any signs of damage. Gefen and Ousey (2020b)	V	B
	18	Wash hands before and after touching mask when outside of areas of patient contact. European Pressure Ulcer Advisory Panel (EPUAP; 2021), Padula et al. (2021)	II	A
	19	It is best to remove all PPE and interface material every 4 h. If the interface material or PPE is wet or damaged, it must be changed immediately. McArthur et al. (2015), Alves et al. (2020), Chen et al. (2020), Coyer et al. (2020), Gefen and Ousey (2020b)	V	B
	20	If possible, switching to PPE with fewer direct contact points, such as face masks that can remain on place using a head band rather than ear loop, may be beneficial. Padula et al. (2021)	V	B
Reasonable use of dressings	21	Use thin prophylactic dressings to prevent pressure injury or protect already injured areas. Alves et al. (2020), American Academy of Dermatology Association (AAD; 2021), European Academy of Dermatology Association (EADV) (2021), European Pressure Ulcer Advisory Panel (EPUAP) (2021), Chen et al. (2020), Coyer et al. (2020), Gasparino et al. (2021); Padula et al. (2021); Steven & Esther (2021), Yu et al. (2021)	V	B
	22	Thin prophylactic dressings can be cut into strips for the nasal bridge, cheek bones and areas behind the ears if in contact with mask or straps. McArthur et al. (2015); Alves et al. (2020); Chen et al. (2020), American Academy of Dermatology Association (AAD; 2021), European Academy of Dermatology Association (EAD; (2021), European Pressure Ulcer Advisory Panel (EPUAP; (2021)	V	A
	23	When using prophylactic dressings, the material–PPE interface should be re‐evaluated on a regular basis to ensure best fit and appropriate skin management. McArthur et al. (2015), Alves et al. (2020), Coyer et al. (2020), European Pressure Ulcer Advisory Panel (EPUAP; 2021)	V	A
	24	The proposed materials are thin foams, film dressings and hydrocolloids. McArthur et al. (2015), Alves et al. (2020), Chen et al., 2020, European Academy of Dermatology Association (EADV) (2021), Coyer et al. (2020), Gasparino et al. (2021), Padula et al. (2021), Yu et al. (2021)	V	B
	25	Stacking multiple dressings is not recommended as it may increase pressure. European Academy of Dermatology Association (EADV) (2021), Gefen & Ousey (2020b), Padula et al. (2021)	V	A
	26	Apply dressings to the skin WITHOUT tension to avoid medical adhesive‐related skin injury. McArthur et al. (2015), American Academy of Dermatology Association (AAD; 2021))	V	A
	27	Facial prophylactic dressings are single‐use only. Coyer et al. (2020)	V	A
treatment measures	28	Abrasions should be treated with topical moisturizers, liquid skin protectants/sealants or cyanoacrylates. European Academy of Dermatology Association (EADV; 2021), European Pressure Ulcer Advisory Panel (EPUAP; 2021), Padula et al. (2021), Yan et al. (2020)	V	B
	29	Thin occlusive dressings may be used to protect open wounds if they do not compromise the mask seal. McArthur et al. (2015), European Academy of Dermatology Association (EADV) (2021), European Pressure Ulcer Advisory Panel (EPUAP) (2021), Yan et al. (2020)	V	B
	30	Deep tissue pressure injury and stage 3, stage 4 and unstageable pressure injuries should be referred for professional wound care. European Pressure Ulcer Advisory Panel (EPUAP) (2021), Padula et al. (2021)	V	B
Education and training	31	Educating healthcare professionals about PPE placement and personal hygiene healthcare facilities should establish clear policies for educating healthcare professionals. Padula et al. (2021)	V	B

## 4 DISCUSSION

### 4.1 Scientific nature of the best evidence for the prevention and management of facial pressure injuries in medical staff

In the JBI Evidence‐based Health Care model, evidence synthesis is a key step in evidence‐based practice, and the summary of evidence for a certain clinical problem and a certain specialty area is an important form of evidence synthesis (Jordan et al., 2018). Therefore, our research team summarized the evidence on the prevention and management of facial pressure injuries in medical staff. These findings can be a scientific basis for preventing and managing PPE‐induced facial pressure injuries in medical staff working on the frontlines of the pandemic. In this research, we followed an evidence‐based methodology, defined the research questions through PIPOST, retrieved the evidence layer by layer according to the ‘6 S’ model and tried to obtain focused and sufficient evidence. The quality of the included studies guarantees high reliability of the evidence. To evaluate the quality of the included studies and to ensure the scientific robustness of the evaluation results, we strictly followed the evaluation process and used the quality evaluation standards for various study types issued by the internationally recognized JBI Evidence‐Based Health Care Center. The makers of this evidence comprise evidence‐based methodology experts and medical staff with anti‐epidemic experience. The evidence included kin cleaning and care, PPE placement and movement, reasonable use of dressings, treatment measures, education and training. In the process of formulating recommendations, we carefully considered the clinical experience and the feelings of medical staff, and good clinical applicability of the findings was accordingly ensured.

### 4.2 Significance of the best evidence summary for the prevention and management of facial pressure injuries for medical staff

Before the COVID‐19 outbreak, most studies aimed at preventing and managing pressure injuries were related to hospitalized patients. It is only during this outbreak that medical staff were acknowledged as the victims of pressure injuries. Generally, the sustained friction generated by the prolonged use of medical devices in hospitalized patients can damage the skin and subdermal tissues (Gefen & Ousey, 2020a; Gefen & Ousey, 2020b). A similar tissue damage manifesting as skin tears or friction damage occurs in medical staff because of the use of PPE, particularly N95 masks and goggles (Peko Cohen et al., 2019). Clinical teams engaged in COVID‐19 treatment continue to report facial skin tears and injuries due to prolonged use of protective masks (Rundle et al., 2020). Once the skin is damaged, it cannot serve as a natural barrier against infection. The area of skin damage can also become a channel for coronavirus and other bacterial, viral and fungal infections (Padula et al., 2021). Therefore, such personnel may become carriers of the virus, causing the risk of spreading infection to other medical staff members and between medical staff and patients (Padula et al., 2021). Furthermore, facial pressure injuries can also cause visible trauma and scars, change the body image and affect the quality of life (Alves et al., 2020). Like we do for hospitalized patients, even for hospital staff, we can reduce the intensity of pressure (and shear force) and reduce the duration of pressure (and shear force) to improve individual tissue tolerance (including the impact of friction and wet environment on tissue tolerance) to prevent and manage facial pressure injuries (European Pressure Ulcer Advisory Panel (EPUAP), 2021). Therefore, we summarized and consolidated the evidence related to this topic. We summarized the five aspects of the prevention and management of facial pressure injuries in medical staff. There are 31 pieces of best evidence. The best evidence extracted in this study was based on expert consensus issued by various societies, systematic reviews and randomized controlled trials, and the overall quality of the evidence in this study was relatively high.

### 4.3 Prevention and management of facial pressure injuries in medical staff

The evidence derived from articles 1 to 14 emphasizes the importance of keeping the skin clean and moisturized. The use of a skin protectant is highly recommended. The following reasons may explain the high incidence of pressure injuries (Chen et al., 2020; Jobanputra et al., 2021; Smart et al., 2020). First, the damage is directly attributed to the pressure and friction caused by PPE. Second, in medical work, medical staff need to compress the metal nose clip of the mask and tighten the elastic rope to ensure appropriate seal of the mask. Such materials with a small contact area and hard texture exert high pressure on local tissues. Third, medical staff wear PPE for a long time during high‐intensity work; this leads to excessive sweating, which is not easy to resolve without removing the PPE. This results in a wet skin surface, consequently resulting in pressure injuries. As medical staff have been under a lot of mental stress and workload during the COVID‐19 pandemic, the skin sweats a lot, which increases the coefficient of friction (COF) between the facial skin and PPE (Gefen & Ousey, 2020a; Gefen & Ousey, 2020b). Excessive moisture and friction cause local damage to the skin barrier function (Kleesz et al., 2012). A study showed that the skin response to respiratory protective equipment (RPE) was characterized by impaired skin barrier function. Excessive skin moisture will lead to sweating, and areas with increased sweating will have high transdermal water loss (Hua et al., 2020). The normal acidic pH of the stratum corneum plays an important role in the formation and maintenance of the permeability barrier and antibacterial defence (Hua et al., 2020). Therefore, keeping the skin clean and moist while using PPE can effectively prevent facial pressure injuries. It has been recommended that a pH‐balanced and ‐insensitive skin cleanser should be used to clean the skin before and after wearing PPE. Scrubbing or massaging the skin with force should be avoided, and the skin should be kept clean and dry. The use of skin barrier protection products is recommended to protect the patient's skin and reduce the incidence of pressure injuries in medical staff.

The evidence derived from articles 15–20 is explained in terms of wearing PPE and mobility. Studies have shown that wearing PPE for a long time is associated with a higher incidence of PPE‐induced pressure injuries (Pittman et al., 2015). However, when treating patients with COVID‐19, medical staff have to wear masks for a long time to avoid infection, which undoubtedly causes continuous pressure on the facial skin (European Pressure Ulcer Advisory Panel (EPUAP), 2021). Therefore, it is imperative that hospital managers formulate practically feasible rules and regulations and adjust the shift timings reasonably. Accordingly, a 4‐h shift with PPE on could be ideal to prevent facial pressure injuries in medical personnel.

The evidence derived from articles 21–27 helps draft recommendations on the selection of protective dressings and the precautions associated with their use. The metal parts and straps of the N95 mask that rest on the bridge of the nose are relatively hard, and the frame of the goggles is hard as well (Friedman et al., 2019; Lei et al., 2012). During use, these hard areas rub against the facial skin and exert a lot of shear force (Gefen & Ousey, 2020b). Therefore, the use of protective dressings on vulnerable parts of the face can reduce the pressure load to a great extent (Peko Cohen et al., 2019). Studies have reported that these protective dressings can be cut in such a way that they can be stuck to the bridge of the nose, cheeks, and behind the ears. They not only provide local cushioning, reduce facial tissue deformation, and mechanical force (European Pressure Ulcer Advisory Panel (EPUAP), 2021; LeBlanc et al., 2021; Peko Cohen et al., 2019) but also reduce skin surface pressure. They also help release trapped heat, which would otherwise cause excessive sweating (require experimental) and related skin irritation and maceration (Amrani et al., 2020; Schwartz & Gefen, 2020).

The evidence derived from articles 28–30 states that if facial skin injuries have occurred, appropriate treatment measures should be taken promptly. As mentioned above, the skin is the body's natural barrier against infection. In this environment, if damage occurs, the damaged area will become a channel for infection of the virus (Padula et al., 2021), which increases the risk of infection and warrants immediate attention.

The evidence derived from article 31 illustrates the importance of providing education and training to medical staff. In fact, there are many sizes of N95 masks which vary with its manufacturer (Desai et al., 2020). PPE discomfort can increase skin damage on the face, ears and scalp and increase the exposure to COVID‐19 (Gefen & Ousey, 2020a, 2020b). Therefore, it is extremely important for medical institutions to train medical staff on proper use of PPE for optimal prevention and management of pressure injuries.

## 5 CONCLUSIONS

Due to the COVID‐19 outbreak, facial pressure injuries in medical staff have become common. These injuries not only cause unpleasant emotional experiences but also increase the risk of infection. Therefore, effective prevention and management of facial pressure injuries are extremely important. We systematically searched for articles on facial pressure injuries of medical workers and summarized 31 pieces of evidence into five aspects: skin cleaning and care, PPE placement and movement, reasonable use of dressings, treatment measures education and training. To ensure safety, clinical medical staff are requested to choose the method best suited to them to prevent facial pressure injuries.

## 6 LIMITATIONS

Some limitations regarding the prevention and management of facial pressure injuries in medical staff need to be acknowledged. Thus far, no practical guidelines on facial pressure injuries in medical staff have been published, and there is a lack of high‐quality randomized controlled studies. Therefore, our study is not included in the guidelines, and only one randomized controlled study is included.

## AUTHOR CONTRIBUTIONS

H.S. conceptualized the study. H.S. and Q. L. wrote the first version. Y. K. reviewed and revised it critically for important intellectual content. H.S., Q.L. and H.Z. searched the database and collected the data. F. Z., B. X. and Q. Z. evaluated the quality of literature. H. S. and Q. L. summarized evidence analysis. H.S. and Q. L contributed equally to this study.

## FUNDING INFORMATION

This study was supported by Fujian Science and Technology Planning Project (2020Y0080) and Military Biosafety Reach Special Project (20SWAQK48) in China.

## CONFLICT OF INTEREST

The authors declare that they have no conflict of interest.
